# Five‐day ski camp could enhance postural stability in young adults: A quasi‐experimental study

**DOI:** 10.14814/phy2.70501

**Published:** 2025-08-25

**Authors:** Áron Horváth, Piroska Béki, Júlia Patakiné Bősze, Ádám Koncz, Attila Szabo, Ferenc Köteles

**Affiliations:** ^1^ Institute of Psychology, Károli Gáspár University of the Reformed Church in Hungary Budapest Hungary; ^2^ Ádám György Psychophysiology Research Group Budapest Hungary; ^3^ Institute of Health Promotion and Sport Sciences, ELTE Eötvös Loránd University Budapest Hungary; ^4^ Faculty of Health and Sport Sciences Széchenyi István University Győr Hungary

**Keywords:** balance, multisensory integration, postural control, postural stability, ski camp

## Abstract

This study investigated whether a 5‐day ski camp could improve postural stability in young adults. It was hypothesized that skiing would reduce postural sway. In this quasi‐experimental design, 43 undergraduate students who participated in a 5‐day ski camp (approximately 20 h of skiing) were compared to 35 peers who did not attend. Postural stability was assessed using the modified Clinical Test of Sensory Integration and Balance protocol of the Balance Tracking System, which evaluates sway under four standing conditions: eyes open or closed, and on stable or unstable surfaces. Quade nonparametric ANCOVAs were used to compare percentage change scores between groups, controlling for age. No significant group differences emerged for standard, proprioceptive, or vestibular postural stability (*p* > 0.05). However, a statistically significant group effect was found for visual postural stability (*p* = 0.006), with improvement observed only in females (*p* = 0.003), not in males (*p* = 0.961). A 5‐day ski camp significantly enhanced visual postural stability in females but did not affect males or other postural domains. These findings suggest a potential sex‐specific adaptation to skiing and highlight the need for further research into the mechanisms underlying balance improvement.

## 1 INTRODUCTION

Interoception, the perception of internal bodily signals, plays a significant role in postural stability by enhancing body awareness and facilitating the integration of sensory information necessary for maintaining balance (Weiniger & Schilaty, 2024). Balance is a crucial aspect of human motor function, relying on the maintenance of stability and postural control. As balance substantially deteriorates with aging (Laughton et al., 2003), maintaining this ability is particularly important given the frequency of falls and related injuries (Osoba et al., 2019). From a sports perspective, postural control is associated with aspects of athletic performance (Paillard, 2019). For example, elite athletes have been shown to possess superior balancing abilities compared to non‐elite athletes in several sports, including rifle shooting, soccer, and golf (Hrysomallis, 2011). Poor balance is a significant predictor of athletic injuries (Romero‐Franco et al., 2014), and balance training has been demonstrated to be an effective means of decreasing injury risk (Hrysomallis, 2007). These results demonstrate that knowing whether a given activity enhances balance ability can be crucial information for healthcare and sports professionals. The goal of the current manuscript is to check whether a 5‐day recreational skiing activity improves postural stability.

The nervous system uses three primary sensory systems for postural control (Goble et al., 2019). First, proprioceptive information provides information about joint position and movement, muscle length, and contraction (Proske & Gandevia, 2012). Second, the visual system provides (head‐centered) information about changes in the external environment. Third, the vestibular system detects the position of the head and its accelerating/decelerating movements in three‐dimensional space (Angelaki & Cullen, 2008). Information from these sensory modalities becomes integrated at the low level of processing, that is, in the superior colliculus in the midbrain (Damasio & Carvalho, 2013). Relying on these sources, the nervous system can adaptively weigh the relative contribution of the three modalities to postural control according to the current situation. Specifically, if one modality does not provide reliable information (for example, if visual information is strongly limited) then the information provided by the other modalities will dominate multisensory integration (Hwang et al., 2014).

For individual testing and experimental purposes, it is possible to modify the brain's relative reliance on different modalities by modifying visual input and the stability of the standing surface (Goble et al., 2019; Shumway‐Cook & Horak, 1986). With eyes open, standing on a stable surface enables the brain to use all three modalities. Closing one's eyes in this situation eliminates visual feedback, and the nervous system relies primarily on proprioceptive information. An unstable surface makes proprioceptive information less reliable. In this situation, with eyes open, visual information dominates, whereas with eyes closed, the vestibular modality takes the primary role in stabilizing posture (Goble et al., 2019).

As mentioned earlier, many sport activities require a high degree of postural control. Skiing is one of them, as maneuvering on skis demands a high level of postural control. According to a study by Andreeva et al. (2021), alpine skiers exhibited reduced postural sway compared to nonathletes. In the same vein, both beginner and intermediate participants demonstrated improved balancing ability after a 7‐day ski camp, as reported in the study by Wojtyczek et al. (2014). However, a 12‐week alpine skiing training program did not improve the postural stability of beginner elderly individuals in Müller et al.'s (2011) study. Additionally, Staniszewski et al. (2016) found that after 9 days of skiing, both beginner and advanced participants showed improvement in postural stability only when wearing ski boots. Overall, while some studies show possible positive effects, the findings are inconclusive.

The goal of the current study was to expand knowledge about the effect of skiing on postural stability in beginner skiers participating in a 5‐day ski camp. We hypothesized that even after such a relatively short time, the activity would improve postural stability, that is, reduce postural sway.

## 2 METHODS

### 2.1 Participants

Seventy‐eight undergraduate students studying recreation science at a large urban university participated in the study (mean age: 20.5 ± 1.4 years, 44 women). The experimental group (*N* = 43, mean age: 20.7 ± 1.4 years, 29 women) consisted of students who participated in a university‐organized ski camp, with participants from two ski camps included in the study. The control group (*N* = 35, mean age: 20.4 ± 1.4 years, 14 women) consisted of fellow students who did not participate, as many chose to participate in a ski camp later. Participants in both groups were beginners, meaning they had no prior experience with skiing. Most participants engage in regular sports; these characteristics are presented in Table 1. Participation in the study was voluntary; students did not receive any reward for their participation. The protocol was approved by the Research Ethics Committee of the Faculty of Pedagogy and Psychology of Eötvös Loránd University (approval number: 2022/12). All participants signed an informed consent form prior to the experiment, agreeing to participate and to the publication of the anonymous group results.

**TABLE 1:** Sporting characteristics of the participants.

		Control group	Experimental group
Training time (M ± SD hours/week)		7.63 ± 6.83	8.48 ± 4.61
Number of participants attending a certain sport	Team sport	17	8
	Strength training	6	13
	Individual sport	4	4
	Dance	2	5
	Combat sport	1	5
	Fitness/wellness	0	2
	Gymnastics	1	2
	No sport	4	3

### 2.2 Measurement of postural stability

To assess postural stability, we used the BTrackS Balance Plate and BTrackS Assess Balance software (Balance Tracking Systems, San Diego, CA). We utilized the modified Clinical Test of Sensory Integration and Balance (mCTSIB) protocol, and the testing and evaluation of performance were conducted according to the prescribed and validated methods and specifications (Goble et al., 2019, 2020). The protocol consisted of four testing conditions. The so‐called standard condition, which relies on visual, proprioceptive, and vestibular information, is performed with eyes open on a stable surface. The proprioceptive condition is performed with eyes closed on a stable surface; the visual condition is performed on an unstable surface with eyes open; finally, the vestibular condition is performed with eyes closed on an unstable surface. Each condition is performed for 20 s. For the unstable surface, a regular (approximate size: 48*40*6 cm) Airex Balance Pad (Airex AG, Sins, Switzerland) was placed on the platform. Participants did not wear shoes during the measurements, and they stood with their feet shoulder‐width apart, keeping their arms on their hips and keeping their heads straight. They stood in front of a wall surface that served as a visual target, with no moving objects/people in front of them. The experimenter warned them about the possibility of losing balance and monitored them to prevent any falls. No incidents of this occurred during the measurements. The sampling frequency was 25 Hz. We have used the Center of Pressure (COP) path length index to assess performance, where higher values indicate worse postural stability. The software calculated the index; for further information, see Goble et al., 2019. Due to technical issues, we had some missing data for each condition (baseline measurement: standard *n* = 5, vestibular *n* = 4, proprioceptive *n* = 4, visual *n* = 3; post‐intervention measurement: standard *n* = 2, vestibular n = 2, proprioceptive *n* = 4, visual *n* = 4). Those with missing values were excluded only from the respective analysis.

### 2.3 Procedure

As measurements were carried out before and after an optional (i.e., voluntarily chosen) ski camp, participants of the study could not be randomly assigned to the experimental (ski) and control (passive control) groups. The ski camp lasted for 5 days; students spent 2 × 2 h with supervised training every day. These sessions focused on developing and practicing fundamental skiing skills. Additionally, participants had the opportunity to practice independently in their free time. Pre‐intervention (baseline) measurements were conducted 5 days prior to the camp. In contrast, post‐intervention measurements were conducted 5 days after the camp (i.e., a total of 14 days elapsed between the two assessments). The control group received no specific intervention during this period. Measurements of the control group were made during the same period. However, a 21‐day gap existed between the two measurements due to technical reasons.

### 2.4 Sample size calculation and statistical analysis

We used G*Power (V. 3) software (Faul et al., 2007) to calculate the minimum sample size required for our statistical analyses. For a multivariate repeated measures analysis of variance (MANOVA) involving two groups and two repeated measures, with a medium effect size (*f* = 0.25), a minimum power (1−*β*) of 0.80, *α* = 0.05, and a 0.5 correlation between repeated measures, the required sample size was 98. Due to opportunistic sampling, our sample size was only 80.61% of the minimum required sample size, prompting us to select a less powerful but less sensitive nonparametric test for the sample size.

## 3 RESULTS

To assess the normal distribution of the data, we conducted both the Kolmogorov–Smirnov and Shapiro–Wilk tests for normality. Statistically significant deviations from normality were found in all four sensory conditions in both tests (all *p* values <0.05), indicating that the assumption of normal distribution was violated. Therefore, the use of nonparametric statistical analyses was also supported by the non‐normal distribution of data (Appendix: Table A1).

### 3.1 Group equality checks at baseline

We calculated Mann–Whitney *U* tests using a Monte Carlo simulation (for more robust *p* values) based on 10,000 sampled tables with starting seed = 2,000,000 to explore baseline comparability between two groups across four measures: standard, proprioceptive, visual, and vestibular. The results showed no significant difference between groups for standard (*U* = 655.00, *p* = 0.991) and visual postural stability (*U* = 643.00, *p* = 0.594). However, proprioceptive postural stability differed between the two groups (*U* = 512.50, *p* = 0.050), with the Monte Carlo estimation confirming the significant difference (*p* = 0.049, 99% CI [0.044, 0.055]). Similarly, a statistically significant difference was found for vestibular postural stability (*U* = 468.00, *p* = 0.026), supported by Monte Carlo results (*p* = 0.025, 99% CI [0.021, 0.029]). These results showed that while two baseline measures were comparable, the other two differed between groups, prompting us to account for these differences in subsequent analyses by calculating the percentage‐difference (Δ) scores.

### 3.2 Sex equality checks at baseline

To examine whether baseline differences in COP path length existed between males and females, Mann–Whitney *U* tests were conducted for each of the four sensory conditions (standard, proprioceptive, visual, and vestibular). The results showed no statistically significant gender differences in the standard condition (*U* = 551.50, *p* = 0.189), the proprioceptive condition (*U* = 528.50, *p* = 0.069), or the visual condition (*U* = 647.00, *p* = 0.549). However, a statistically significant difference was observed in the vestibular condition (*U* = 494.00, *p* = 0.034), with females (mean rank = 44.03) exhibiting higher COP path length values than males (mean rank = 33.26).

These findings suggest that, although most baseline measures were comparable between the sexes, the vestibular condition exhibited a statistically significant gender difference. Therefore, we considered using percent difference (Δ) scores as an appropriate approach, as they help control for this baseline inequality in the subsequent analyses.

### 3.3 Percent difference (delta [Δ]) score calculations

When baseline values differ between groups, simply calculating difference scores (post‐test minus pre‐test) does not fully account for these initial inequalities because the absolute change does not consider the starting point. In contrast, calculating percent difference scores ([post−pre]/pre * 100) normalizes the change relative to the initial value, ensuring that differences are proportionally scaled. This method enables a fairer comparison of change scores (Δ scores) by accounting for baseline variability, thereby reducing the risk of misinterpreting results due to unequal starting points. Consequently, we used the percent Δ scores in the subsequent tests. In this case, negative values refer to a decrement in COP, thus indicating better postural stability.

### 3.4 Correlations

Given the significant correlations between age and the standard (*ρ* = 0.275, *p* = 0.019) and proprioceptive (*ρ* = 0.269, *p* = 0.022) measures (Table 2), as well as the significant correlation between sex and the visual measure (*ρ* = −0.322, *p* = 0.005), it is necessary to control for age and sex to isolate the effects of the grouping factor on the dependent variables.

**TABLE 2:** Spearman rank correlations (rho [*ρ*] coefficients) between sex, age, and percent change scores in four measures.

Correlations						
		Age	Sex	1	2	3
Sex	*ρ*	−0.061	–			
	*p*	0.591				
Standard (1)	*ρ*	0.275^a^	−0.141	–		
	*p*	0.019	0.237			
Visual (2)	*ρ*	−0.017	−0.322^b^	0.294^a^	–	
	*p*	0.888	0.005	0.015		
Proprioceptive (3)	*ρ*	0.269^a^	0.044	0.014	−0.173	–
	*p*	0.022	0.711	0.912	0.162	
Vestibular (4)	*ρ*	−0.162	0.036	0.078	−0.012	0.112
	*p*	0.168	0.762	0.529	0.925	0.366

^a^ Correlation is significant at the 0.05 level (2‐tailed).

^b^ Correlation is significant at the 0.01 level (2‐tailed).

### 3.5 Group comparisons

Quade nonparametric analyses of variances (ANCOVAs) were conducted to compare the two groups on the percent Δ scores (Table 3), with age included as a covariate, as this method adjusts for age‐related variability while accounting for potential differences in the relationship between age and the dependent variable across the two groups, ensuring accurate group comparisons without assuming equal regression slopes. For standard postural stability, the main effect for groups, after controlling for age, was statistically not significant (*F* (1, 69) = 2.54, *p* = 0.116). Similar nonsignificant results emerged for proprioceptive (*F* (1, 70) = 0.48, *p* = 0.492) and vestibular postural stability (*F* (1, 71) = 1.88, *p* = 0.164). However, statistically significant differences were observed for visual postural stability (*F* (1, 70) = 7.94, *p* = 0.006).

**TABLE 3:** Descriptive statistics for percent delta (Δ%) COP path length by group and sensory condition.

	Control group			Intervention group		
	Median	Mean	Standard deviation	Median	Mean	Standard deviation
Standard	1.33	6.82	25.37	−2.03	2.62	29.82
Visual	2.68	4.83	17.49	−12.60	−3.89	34.05
Perceptual	−3.11	1.40	30.17	1.49	8.27	31.92
Vestibular	−1.72	2.32	34.26	6.75	12.98	45.26
Males						
Standard	−1.64	4.72	20.63	11.50	11.50	34.29
Visual	7.82	6.38	19.16	−2.95	11.75	36.29
Perceptual	1.97	6.12	35.74	−2.04	5.80	42.81
Vestibular	−3.66	−2.17	30.36	6.80	23.42	65.64
Females						
Standard	6.15	9.37	30.80	−4.55	−2.17	26.61
Visual	−2.90	2.70	15.37	−13.60^a^	−10.37	31.47
Perceptual	−5.57	−5.86	17.63	10.27	9.36	26.65
Vestibular	0.00	8.53	39.47	6.32	7.77	30.85

*Note*: Values represent the median, mean, and standard deviation for each condition in the Control and Intervention groups, as well as separately for males and females. Negative values indicate a reduction in COP path length, interpreted as improved postural stability.

^a^ Indicates a statistically significant difference between the control and intervention groups.

**FIGURE 1:**
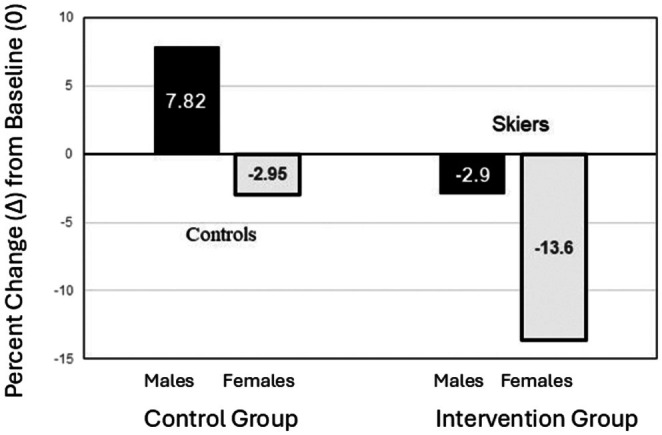
The median (in the center of bars) of the percent change (Δ) scores in visual postural stability from pre‐ to post‐skiing in males (black bars) and females (gray bars) in control (left) and skiing (right) groups. The line at 0 is the baseline before the skiing camp started.

Given that sex was statistically significantly correlated with visual postural stability, and the results also revealed statistically significant group main effects on this measure, we performed another Quade ANCOVA separately for males and females. These results revealed that group differences were statistically significant for females (*F* (1, 40) = 10.32, *p* = 0.003) but not for males (*F* (1, 28) = 0.002, *p* = 0.961). To follow up on these results, we conducted Mann–Whitney *U* tests comparing the intervention and control groups on percent difference (Δ%) scores separately for females. A statistically significant difference was found in the visual condition (*U* = 76.00, *p* = 0.002), with the intervention group showing greater improvement in postural stability. No significant differences were observed in the standard (*U* = 130.00, *p* = 0.140), proprioceptive (U = 114.00, *p* = 0.076), or vestibular (*U* = 177.00, *p* = 0.889) conditions. These results reinforce the finding that the intervention significantly improved visual postural stability among females. The results are illustrated in Figure 1.

## 4 DISCUSSION

In a quasi‐experiment involving 78 young individuals with no prior skiing experience, a 5‐day skiing camp improved postural stability in the visual condition for females but not for males. No improvement was found in the standard, proprioceptive, and vestibular conditions for either males or females. The possible association between skiing and postural stability is not as straightforward as it seems at first sight. Table 4 presents a summary of the most important aspects and results of the studies that we are aware of on this topic.

**TABLE 4:** Summary table of studies conducted on postural stability and skiing.

Author, year^a^	Sample	Research design	Postural stability measurement conditions	Result
Noé and Paillard (2005)	National (*n* = 7) and regional (*n* = 7) level skiers	Cross‐sectional comparison	Eyes open/eyes closed, stable/unstable surface, with/without ski boots	Better performance of regional skiers without ski boots
Müller et al. (2011)	Elderly individuals divided into intervention (*n* = 27) and control group (*n* = 20)	Effect of a 12‐week intervention (28.5 days of guided skiing)	Unstable surface, eyes open	No improvement
Wojtyczek et al. (2014)	Beginner (*n* = 43) and intermediate (*n* = 35) university students	Effect of a 7‐day ski camp (31.5 h of skiing)	Unstable surface, eyes open	Improvement in both groups
Staniszewski et al. (2016)	Beginner (*n* = 10) and advanced *n* = 10) university students	Effect of a 9‐day ski camp	Stable surface, eyes open/eyes closed, with/without ski boots	Improvement only in ski boots
Andreeva et al. (2021)	Alpine skiing and snowboarding athletes (*n* = 49) and nonathletes (*n* = 225)	Cross‐sectional comparison	Stable surface, eyes open/eyes closed	Better performance with eyes open
Current study	Beginner university students (*N* = 79)	Effect of a 5‐day ski camp (20 h of skiing)	Stable/unstable surface, eyes open/closed	Improvement with eyes open on unstable surface, but only for women

^a^ In chronological order.

Several factors can influence the outcome of studies exploring this area of research. For example, it was shown that the balancing ability cannot be generalized. Static balance refers to the condition in which the platform remains undisturbed, whereas dynamic balance involves maintaining posture when the base of support is displaced or external stimuli are applied (Rizzato et al., 2021). Interestingly, no significant association was found between performance in the two types of tasks (Rizzato et al., 2021). Every study in Table 4 used static balance as an outcome variable. However, the tasks differed depending on whether participants stood on a stable or unstable surface, had their eyes open or closed, and wore ski boots. Staniszewski et al. (2016) showed improvement only in ski boots after a 9‐day ski camp. Comparably, Müller et al. (2011) found no improvement after a 12‐week ski camp in elderly individuals, as measured by postural stability without ski boots. Wojtyczek et al. (2014) finding contradicts this pattern, reporting an improvement in balancing ability after a 7‐day ski camp in university students. A possible explanation for this result is that the postural stability task they applied may require more similar corrective movements to those involved in skiing. The cross‐sectional study of Noé and Paillard (2005) showed that regional‐level skiers perform better than national‐level skiers without ski boots. The cross‐sectional comparison by Andreeva et al. (2021) revealed that alpine skiing and snowboarding athletes possess better balance abilities than nonathletes. However, this finding might reflect the general beneficial effect of sport on balance.

The current study extends these findings, showing that the improvement might not only be test‐specific but can also depend on the sex of the participants. Skiing not only enables but requires the use of visual information. Skiers need visual acuity, contrast sensitivity, peripheral vision, and fast processing to be able to navigate effectively and safely (Očić et al., 2022; Stalin et al., 2021; Stalin & Dalton, 2021). Also, navigating on skis resembles navigating an unstable surface, which may be the reason for improvement only in the visual condition. The sex‐specific improvement might be due to differences in multisensory integration. Namely, women are more likely to rely on visual information (Barnett‐Cowan et al., 2010), which may lead them to develop more expertise in visual tasks. It should also be noted that our participants were novices in skiing, implying that a substantial portion of the training time was dedicated to acquiring and practicing fundamental skills. Consequently, the observed results may be attributable not only to five consecutive days of skiing but also to the process of learning to ski. While our findings suggest that a reduction in COP path length reflects improved postural stability, it is essential to note that this interpretation is not without controversy. Some researchers argue that a shorter path length may indicate increased stiffness or reduced adaptability of the postural control system, potentially representing a compensatory rather than an optimal strategy (Prieto et al., 1996; Winter, 1995). Furthermore, postural sway is influenced by various factors, including attentional focus, task constraints, and neuromuscular control strategies, which indicate that reduced sway does not always equate to better postural stability (Duarte & Sternad, 2008). Therefore, although our results align with studies supporting COP path length as a marker of postural stability improvement (Palmieri et al., 2002), future research should consider multidimensional interpretations of postural stability and incorporate complementary measures such as sway area, velocity, and nonlinear analyses to obtain a more precise and more comprehensive understanding.

This study is not without limitations. For example, the sample consisted of only young individuals (university students), so the findings may not be generalized to other populations. It is also important to note that we have assessed postural stability in static circumstances. However, skiing requires dynamic control. Using a dynamic balancing task would probably reveal more pronounced effects. Additionally, we employed a quasi‐experimental design, which means that participants were not randomly assigned to the groups, whether they chose to participate in the ski camp or not.

One strength of the study is that it assesses postural stability using four different test variations (standard, proprioceptive, visual, and vestibular), which enhances the generalizability of the findings. Additionally, the findings have important practical implications and refine the understanding in this area of research. Based on the results, a recreational ski camp is mostly beneficial for females, particularly with respect to a specific visual aspect of postural stability.

## 5 CONCLUSION

This study highlights that a 5‐day ski camp improved visual postural stability in females but had no significant effects on males or in other conditions. These findings suggest that skiing may enhance postural stability through visual reliance, potentially due to its demands for navigating an unstable surface. The sex‐specific effect may stem from differences in multisensory integration, as women are more likely to depend on visual cues for postural control. Study design and task variations play a crucial role in balance research, emphasizing the need for standardized methods better to understand the relationship between skiing and postural control. Future research should explore how individual factors, such as sex, training duration, and testing conditions, influence balance adaptations in skiing.

## AUTHOR CONTRIBUTIONS

Áron Horváth was involved in conceptualization, methodology, writing—original draft, and investigation. Piroska Béki and Júlia Patakiné Bősze were involved in conceptualization and resources. Ádám Koncz was involved in conceptualization and project administration. Attila Szabo was involved in conceptualization, formal analysis, and writing—review and editing. Ferenc Köteles was involved in conceptualization, methodology, writing—review and editing, and supervision.

## FUNDING INFORMATION

This research was supported by the Research Fund of the National Research, Development, and Innovation Office (K 147788).

## CONFLICT OF INTEREST STATEMENT

The authors declare no conflicts of interest.

## ETHICS STATEMENT

The study was approved by the Research Ethics Committee of the Faculty of Pedagogy and Psychology of Eötvös Loránd University (No: 2022/12).

## PARTICIPANT CONSENT STATEMENT

All participants provided consent for their participation and the publication of anonymous group results.

## Data Availability

Data are available from the first author upon reasonable request.

## Appendix

**TABLE A1:** Data normality tests of dependent measures.

Condition	K‐S statistic	K‐S *p* value	S‐W statistic	S‐W *p* value
Standard	0.116	0.048	0.938	0.005
Visual	0.180	<0.001	0.886	<0.001
Proprioceptive	0.121	0.031	0.914	<0.001
Vestibular	0.121	0.030	0.806	<0.001

Abbreviations: K‐W, Kolmogorov–Smirnov test; S‐W, Shapiro–Wilks test.
